# Oral Cell-Targeted Delivery Systems Constructed of Edible Materials: Advantages and Challenges

**DOI:** 10.3390/molecules27227991

**Published:** 2022-11-17

**Authors:** Xiaolong Li, Zihao Wei, Changhu Xue

**Affiliations:** 1 College of Food Science and Engineering, Ocean University of China, Qingdao 266003, China; 2 Laboratory of Marine Drugs and Biological Products, Pilot National Laboratory for Marine Science and Technology, Qingdao 266237, China

**Keywords:** nutraceuticals, cell targeting, oral administration, biosafety, gastrointestinal tract, mucosal barrier

## Abstract

Cell-targeted delivery is an advanced strategy which can effectively solve health problems. However, the presence of synthetic materials in delivery systems may trigger side effects. Therefore, it is necessary to develop cell-targeted delivery systems with excellent biosafety. Edible materials not only exhibit biosafety, but also can be used to construct cell-targeted delivery systems such as ligands, carriers, and nutraceuticals. Moreover, oral administration is the appropriate route for cell-targeted delivery systems constructed of edible materials (CDSEMs), which is the same as the pattern of food intake, resulting in good patient compliance. In this review, relevant studies of oral CDSEMs are collected to summarize the construction method, action mechanism, and health impact. The gastrointestinal stability of delivery systems can be improved by anti-digestible materials. The design of the surface structure, shape, and size of carrier is beneficial to overcoming the mucosal barrier. Additionally, some edible materials show dual functions of a ligand and carrier, which is conductive to simplifying the design of CDSEMs. This review can provide a better understanding and prospect for oral CDSEMs and promote their application in the health field.

## 1. Introduction

Cell-targeted delivery systems have been successfully constructed and used to solve health problems [1,2]. It is well known that cell targeting can be achieved by the interaction between ligands on the delivery system and receptors on the cell surface, which enhances the therapeutic efficacy of the drug and decreases the off-target cytotoxicity of the drug. For example, polymer nanoparticles modified by galactose enhanced the targeted delivery of siRNA to macrophages, which effectively downregulated the expression of TNF-α mRNA [3]. However, the development of cell-targeted delivery systems constructed of synthetic materials may be limited due to the use of organic solvents and chemical reagents during the production and application of synthetic materials. Furthermore, the long-term administration of drugs (antibiotics, corticosteroids, immunomodulators, and so on) may trigger side effects, such as abdominal pain, diabetes, and osteoporosis [4]. Therefore, it is necessary to improve the biosafety of cell-targeted delivery systems.

The edible materials, such as proteins, polysaccharides, lipids, polyphenols and so on, are the candidates to construct cell-targeted delivery systems overcoming the disadvantages of synthetic materials. The edible materials can be degraded by various enzymes in the body showing excellent biosafety. Additionally, edible materials, which exhibit different molecular weights, solubilities, surface charges, and hydrophilicity/hydrophobicity, can be utilized to construct various delivery systems, such as nano- or microparticles, liposomes, emulsions, and so on. The delivery systems are beneficial to improving the solubility and stability of nutraceuticals [5]. Nutraceuticals are defined as the food or the part of food exhibiting healthy benefits, which are beneficial to the prevention and treatment of diseases. Nutraceuticals, such as polysaccharides, proteins, polyphenols, and so on, are beneficial to health. They play multiple functions in the body, such as anti-oxidation, anti-inflammation, anti-biosis, and so on. For instance, curcumin, as a kind of natural diketone compound extracted from the rhizome of *Curcuma longa*, shows excellent capabilities of anti-inflammation and anti-oxidation, which is positive to the treatment of some diseases, such as tumors and inflammatory bowel disease [6,7,8]. In addition, some edible materials, which are used as ligands, can be combined with cell surface receptors to mediate the cell-targeted delivery [9,10]. Therefore, the ligand, carrier, and therapeutic agent of cell-targeted delivery systems can be entirely comprised of edible materials.

The cell-targeted delivery systems constructed of edible materials (CDSEMs) cannot play a role until they arrive at specific cells. In the same way as food intake, oral administration is a good choice for CDSEMs. Oral administration is the most common strategy for the administration of medication, which can effectively avoid the pain of injection and make patients have good compliance [11,12]. In particular, patients with chronic diseases need to take drugs for a long time or even for life to manage diseases. However, CDSEMs are sensitive to the harsh gastrointestinal environment. Before reaching the targeted cell, CDSEMs are prone to disassembling prematurely under the actions of stomach acid and digestive enzymes, which is one of the greatest challenges [13]. In addition, the diffusion of CDSEMs is limited by the mucosal barrier, which prevents delivery systems from reaching the apical surface of cells. Although polyethylene glycol can act as an excellent material to enhance the mucus penetrability of delivery systems, it can cause an allergic reaction [14,15]. Therefore, it is very important to make full use of the functional properties of edible materials to overcome the challenges of oral administration and take advantage of oral administration.

In this review, the advantages and challenges of oral CDSEMs are discussed. Subsequently, the structure and function of existing oral CDSEMs are comprehensively summarized. Finally, the future perspectives of oral CDSEMs are systematically analyzed, which is beneficial to their application in the health field.

## 2. Advantages of Oral CDSEMs

**Figure 1:**
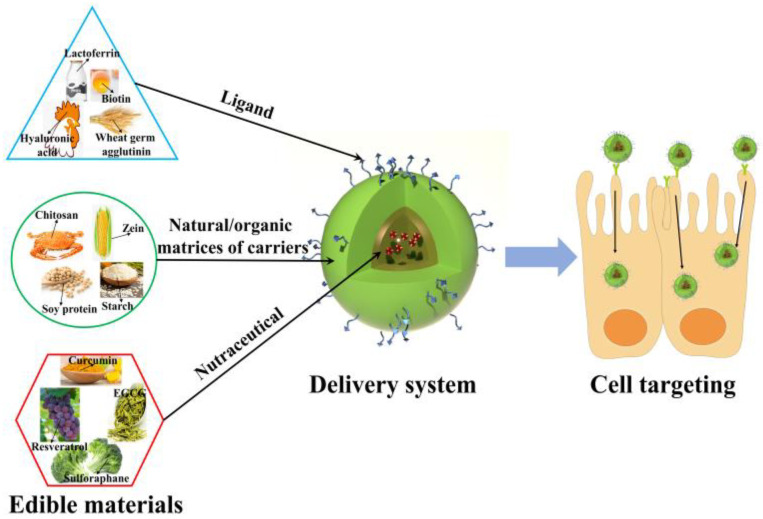
Structure diagram of cell-targeted delivery systems constructed of edible materials such as a ligand, carrier, and nutraceutical.

The combination of the advantages of edible materials and the advantages of cell-targeted delivery technology further enhances the progressiveness of the delivery system. For example, edible materials can act as ligands, carriers, and nutraceuticals to construct delivery systems realizing cell targeting (Figure 1). The ligands, which can be combined with ca ell surface receptor, endow delivery systems with cell-targeted ability [9]. The carrier is used to encapsulate nutraceuticals overcoming their disadvantages of poor solubility and stability. Furthermore, the carrier surface also provides grafting sites for ligands. The nutraceuticals as the cargo can be released to specific cells to maintain the homeostasis. The advantages of oral CDSEMs include the biosafety of raw materials, simplicity of administration, and precision of delivery.

### 2.1. Advantages of Edible Materials

The nutraceuticals can be encapsulated in various delivery systems, which can improve their solubility, stability, and cell-targeted ability [16,17]. However, delivery systems are usually made up of synthetic materials, which requires a lot of time and economic investments to prove their biosafety. Compared with synthetic materials, edible materials are easier to obtain and have better biodegradability and biocompatibility [18]. There are many kinds of edible materials, such as proteins, polysaccharides, lipids, and so on, which have been used to fabricate various delivery systems, such as nano- or microparticles, liposomes, emulsions, and so on [6,19]. These systems showed excellent effects on the encapsulation and delivery of nutraceuticals.

Furthermore, there are many edible materials showing the capability of cell targeting, which can act as the ligands of cell-targeted delivery systems [17,20]. Different ligands can be combined with different receptors expressed on the cell to achieve cell targeting. Therefore, the cell-targeted delivery systems can be fully constructed of edible materials, which endows the delivery systems with outstanding biosafety.

### 2.2. Advantages of Oral Administration

Injection and oral administration are the common forms of administration. Compared with injection, oral administration is the favored way because it has the advantages of noninvasiveness, low cost, and administration simplicity [21]. Oral CDSEMs cannot increase the psychological burden of the patient because CDSEMs are completely composed of edible materials, which is as simple as eating snacks. Compared with the injection products, the production and consumption of oral products have lower costs. For example, the production of an oral product does not require a sterile environment. The administration of oral products does not require professional technicians and sterile syringes. Moreover, the villi, which are the finger-like projections in the small intestine, effectively increase the surface area of the small intestine, providing a wide range of recognition sites for cell-targeted delivery systems. Therefore, oral administration is a good choice to take CDSEMs.

### 2.3. Advantages of Cell Targeting

The efficacy of nutraceuticals depends on their bioavailability rather than intake. Cell-targeted delivery is an effective strategy to improve the bioavailability of nutraceuticals. The nutraceuticals encapsulated in traditional delivery systems can be transported to the intestinal lumen, which increases their bioaccessibility [6]. However, the nutraceuticals encapsulated in cell-targeted delivery systems are transported to specific cells, which can enhance their bioavailability. Cell targeting promotes the accurate delivery of nutraceuticals through the ligand–receptor interaction [22]. Compared with a traditional delivery system, a cell-targeted delivery system is a more intelligent carrier, which can specifically deliver nutraceuticals to cells, fully improve the accumulation concentration of nutraceuticals, and effectively relieve different symptoms.

## 3. Challenges of Oral CDSEMs

Despite oral CDSEMs exhibiting many advantages, many challenges should be overcome to achieve the objective of cell targeting. CDSEMs exhibit excellent biosafety, which also means that they are susceptibly hydrolyzed by enzymes during oral administration. The change in pH in the gastrointestinal tract also places the delivery systems at risk of dissociation. Even if the delivery systems can remain stable in the gastrointestinal tract, the mucus layer of the gastrointestinal tract hinders the interaction of ligands and receptors. Therefore, when the delivery systems are targeted to specific cells through oral administration, they must tolerate the harsh gastrointestinal environment (low pH and enzymatic hydrolysis) and then overcome the mucosal barrier located at the apical surface of cells.

### 3.1. Harsh Gastrointestinal Environment

Oral administration is a good way to play a role in the cell targeting of delivery systems constructed of edible materials, but oral administration is also confronted with great challenges due to the harsh gastrointestinal environment, which greatly affects the stability of delivery systems [12]. The main challenges are the strong acidic environment in the stomach and the presence of digestive enzymes in the gastrointestinal tract [22]. The pH of the stomach lumen ranges from 1 to 2 due to the presence of gastric acid. The proteins are very sensitive to pH and can denaturate in an acidic environment, which affects their function as the carrier or ligand. In addition, the pepsinogen is conversed to pepsin under the action of gastric acid to disintegrate proteins. Therefore, the enzymatic hydrolysis can influence the functions of the carrier and ligand composed of protein, causing the sudden release of nutraceuticals in the stomach or the loss of cell-targeting ability of carriers.

The pH changes from 2 to 7 when the delivery systems are transported into the small intestine from the stomach, which can affect the structure of the protein. The change in protein structure may result in the release of nutraceuticals, which cannot be delivered to the targeted cell. In addition, a large number of digestive enzymes in the small intestine, such as protease, lipase, amylase, and so on, can decompose proteins, fats, and polysaccharides into amino acids, free fatty acids, and glucoses, which can destroy the delivery systems constructed of edible materials. If the targeted cells are located in the colon, it must be considered to design an anti-digestible shell to avoid the premature degradation of the cell-targeted delivery systems in the gastrointestinal tract. In addition, colon-targeted delivery can be realized through the decomposition of the protective shell by the intestinal microbiota. The anti-digestible shell can be utilized by the intestinal microbiota, which is beneficial to health.

### 3.2. Mucosal Barrier

Reaching the apical surface of cells to achieve cell targeting also requires overcoming the mucosal barrier. The mucosal barrier, which mainly consists of mucins secreted by goblet cells, has a reticular structure with a pore size of 10–200 nm, limiting the diffusion of the carrier [23]. The size of the carrier is negatively correlated with the mucus penetrability: a smaller particle is beneficial to the improvement in mucus penetrability [24]. In addition, the hydrophobicity and negatively charged groups of mucins exhibit influences on overcoming the mucosal barrier through hydrophobic and electrostatic interactions, respectively. For example, the enhancement of hydrophobic and electrostatic interactions between the carrier and mucus is conductive to prolonging the retention time of the carrier in the mucus layer, promoting the release of nutraceuticals. The decrease in hydrophobic and electrostatic interactions between the carrier and mucus is conductive to enhancing the mucus penetrability of the carrier.

Therefore, there are two strategies to overcome the mucosal barrier: mucosal adhesion and mucosal penetration [25,26]. Mucosal adhesion is used to prolong the retention time of the carrier in the mucus layer by increasing the interaction between the carrier and the mucus network. Chitosan is the most common material to enhance the mucosal adhesion of the carrier due to its cationic properties. Chitosan was coated on calcium phosphate nanoparticles using the layer-by-layer method, which was beneficial to the enhancement of mucosal adhesion prolonging the retention time of the carrier in the mucus layer [27]. The method of mucosal penetration is used to weaken the interaction between the carrier and the mucus network so that the carrier can quickly penetrate the mucus layer to reach the apical surface of cells. Furthermore, the thickness of the mucus layer in the small intestine and colon is different. The small intestine epithelium is covered by a loose mucus layer, while the colon epithelium is covered by two mucus layers, including a dense mucus layer at the inner surface and a loose mucus layer at the outer surface [28]. The mucus barrier should be sufficiently overcome when delivery systems are targeted to colon cells. The surface structure and morphology of carrier can be further designed to overcome the mucosal barrier. The chitosan derivative nanoparticles with positive charge promoted the mucosal adhesion through the electrostatic interaction with negatively charged mucin, which is beneficial to the targeted delivery of nutraceuticals [26]. A short nanotube composed of α-lactalbumin peptides exhibited outstanding mucus permeability due to its rotation and conformational adjustment ability, which improved the bioavailability of curcumin and was beneficial to the therapy of ulcerative colitis [23].

## 4. Existing Oral CDSEMs

**Figure 2:**
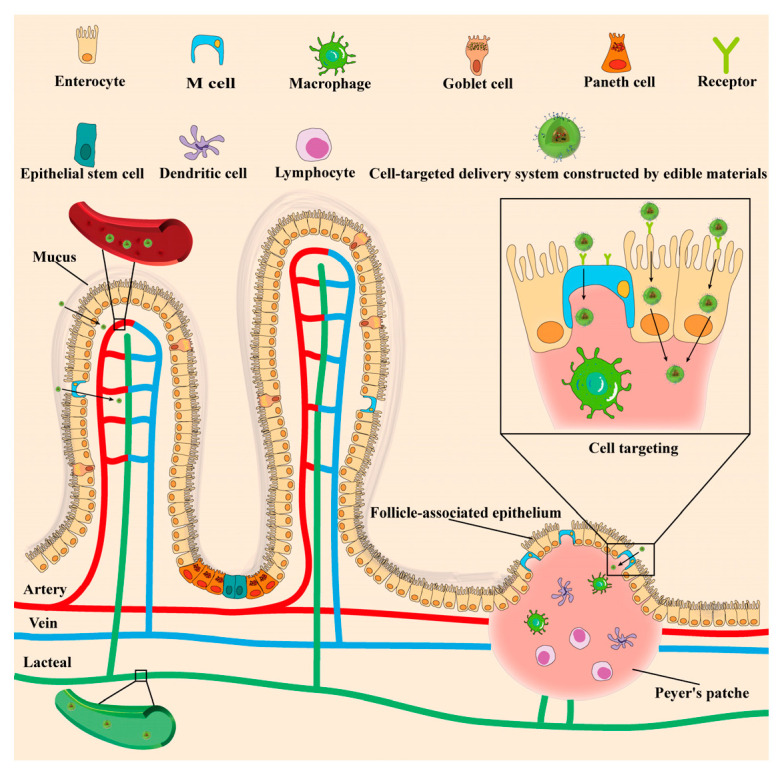
The physiological structure of the intestinal tract and schematic diagram of cell targeting. Adapted with permission from Ref. [30]. Copyright 2018, American Chemical Society.

The intestinal tract is composed of various cells with different functions and characteristics. The cells become the target of oral CDSEMs. The intestinal cells include enterocytes, microfold cells (M cells), goblet cells, Paneth cells, enteroendocrine cells, epithelial stem cells, dendritic cells, macrophages, and lymphocytes, which are located at different locations and play different roles (Figure 2). There are diverse receptors expressed on the surface of the cell. It is well known that the cell targeting of a delivery system is achieved through the ligand–receptor interaction [29]. Therefore, the presence of a ligand is necessary for the cell-targeted delivery systems. In the following sections, the cells, which are targeted by the delivery systems constructed of edible materials, are discussed, such as an enterocyte, macrophage, M cell, dendritic cell, and tumor cell. The structure and function of oral CDSEMs are summarized in Table 1.

### 4.1. Enterocyte Targeting

**Figure 3:**
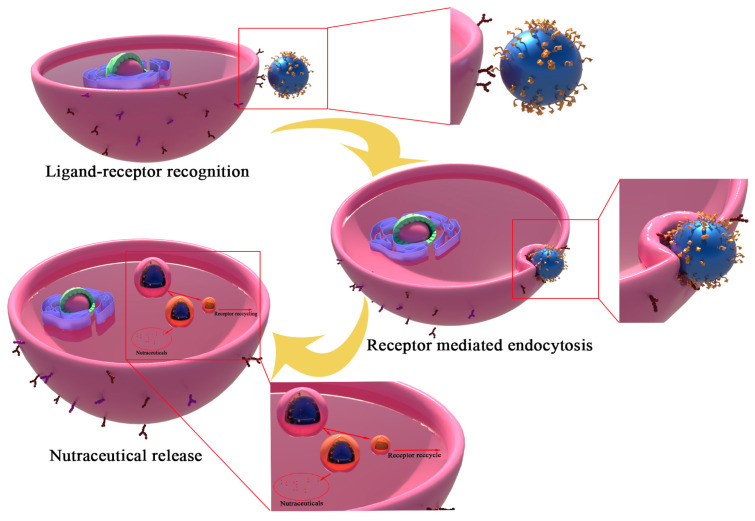
Receptor-mediated endocytosis and the cell-targeted delivery of nutraceuticals.

Enterocytes, which are the most common cells in the intestinal tract, have brushed edges that expand the surface area resulting in the efficient absorption of substances from the intestinal lumen [48]. The delivery systems can be internalized by a specific cell through receptor-mediated endocytosis (Figure 3). Therefore, the interaction, which is based on the receptor of the cell and the ligand of the delivery system, promotes the targeted delivery of nutraceuticals. Different receptors are expressed on the apical plasma membrane of enterocytes, so there are corresponding ligands of enterocytes including vitamins [27], proteins [34], and polysaccharides [35] to mediate the cell-targeted delivery of the carrier.

#### 4.1.1. Vitamin-Based Targeting

A vitamin is a natural and safe substance, which widely exists in food and plays a vital role in the process of growth and development. Some vitamins, which act as ligands, have appeared in oral CDSEMs because of their ability of cell targeting, such as cobalamin (vitamin B_12_) [31] and biotin (vitamin B_7_) [32].

Vitamin B_12_ (VB_12_), mainly found in meat, is the only vitamin containing metal and can act as a ligand to realize the cell-targeted delivery of nutraceuticals [49]. However, VB_12_ should be processed in the gastrointestinal tract to realize cell-targeted delivery during oral administration [49]. Firstly, VB_12_ binds with the R-binder in the stomach. Subsequently, VB_12_ can form the complex with an intrinsic factor (IF), which is a kind of mucin secreted by gastric mucosa. The complex can be combined with an IF receptor expressed on the cell surface to mediate endocytosis. Therefore, the delivery systems modified by VB_12_ can promote the internalization of enterocytes and the targeted delivery of nutraceuticals to enterocytes. Scutellarin, as a bioactive material, can improve vascular endothelial cell dysfunction. However, scutellarin shows low water solubility and bioavailability, limiting its application in the health field [50]. Amphiphilic chitosan modified by VB_12_ interacts with scutellarin through a hydrophobic interaction to form nanoparticles, which are used to promote cell-targeted delivery [31]. The transport volume of scutellarin increased 4.39 times based on the interaction between VB_12_ and the IF receptor. The retinal damage of diabetic rats was relieved by the oral administration of scutellarin-loaded nanoparticles functionalized by VB_12_. The presence of VB_12_ enhanced the cell-targeting ability of the delivery system and the bioavailability of scutellarin, which demonstrated that VB_12_ was a suitable ligand.

Biotin, as an exogenous micronutrient, is involved in metabolism and also promotes protein synthesis, which is important for the growth of cells. The sodium-dependent multivitamin transporters are expressed on the enterocyte surface and responsible for recognizing biotin, which indicates that the biotin can be used as the ligand of delivery systems. Biotin, inulin, and vitamin E were used to construct CDSEMs [32]. Biotin and vitamin E were grafted onto inulin to form a conjugate, which self-assembled into nanomicelles without cytotoxicity. Compared with inulin–vitamin E nanomicelles, the nanomicelles modified by biotin could be transported more quickly by cells, which was due to the ligand–receptor interaction. The biotin, as a natural ligand, endows the delivery system with cell-targeting ability, which promotes the nutraceutical accumulation in the targeted cell.

#### 4.1.2. Lactoferrin-Based Targeting

Lactoferrin, which belongs to the transferrin family, is an iron-binding glycoprotein. It not only has good anti-bacterial and anti-oxidative capacities, but also can be used as a ligand to endow delivery systems with the ability of cell targeting [51]. The effect of the lactoferrin shell on the oral delivery of zein nanocarriers was studied [34]. The zein/lactoferrin nanoparticles were fabricated through the antisolvent precipitation method, in which the hydrophobic core was composed of zein and the hydrophilic shell was composed of lactoferrin. The presence of a lactoferrin shell is beneficial to the cell-targeted delivery of nanoparticles. Compared with zein nanoparticles, zein/lactoferrin nanoparticles could be more effectively uptaken by cells. After the cell was treated with free lactoferrin, the uptake of the cell to the zein/lactoferrin nanoparticles was weakened, which indicated that endocytosis was mediated by the lactoferrin receptor. Furthermore, the cell uptake was enhanced through nonspecific endocytosis mediated by the electrostatic interaction of zein/lactoferrin nanoparticles with glycocalyx. The protective shell should be applied to protect the delivery carriers before reaching the targeted cells. Rhein-loaded lactoferrin nanoparticles were decorated with calcium pectinate and hyaluronic acid (HA) through an electrostatic interaction [33]. The nanoparticles could stably pass through the gastrointestinal tract due to the presence of a calcium pectinate shell. Furthermore, the nanoparticles were specifically released in the colon because the calcium pectinate was decomposed by intestinal microbiota. Lactoferrin played the dual role of carrier and ligand to promote the targeted delivery of rhein to enterocytes, which up-regulated the expression of ZO-1 and Claudin-1 alleviating intestinal injury. In addition, the shell of calcium pectinate not only promotes the colon-targeted release of the carrier, but also may regulate the composition of intestinal microbiota, which is beneficial to the treatment of ulcerative colitis.

#### 4.1.3. Arginine–Glycine–Aspartate (RGD)-Based Targeting

RGD is the ligand of integrin, which can mediate enterocyte targeting. Integrin is a kind of transmembrane glycoprotein expressed on the cytomembrane, which is composed of an α-subunit and a β-subunit. The α-subunit and β-subunit have 18 and 8 variants, respectively, which constitute 24 known integrin subtypes [52]. Different integrin subtypes exhibit different functions. For example, the expression of integrin αvβ3 is related to the growth and invasion of tumors [53]. The integrin αvβ6 is related to epithelial barrier function [54].

It was found that silk fibroin derived from *Antheraea pernyi* contained abundant RGD fragments, which was conducive to the construction of cell-targeted delivery systems [55]. However, the silk fibroin derived from *Bombyx mori* has no RGD fragments, which indicates that not all silk fibroin contains RGD and it depends on the species of silkworm. The *Antheraea pernyi* silk fibroin nanoparticles were fabricated for the targeted delivery of resveratrol [41]. The chitosan–alginate hydrogels were used to enhance the gastrointestinal stability of nanoparticles during oral administration. Compared with silk fibroin nanoparticles without RGD, the internalization of the cell to *Antheraea pernyi* silk fibroin nanoparticles increased 1.7-fold after 2 h, which was due to the interaction between RGD and integrin. Additionally, the α-helix structure of *Antheraea pernyi* silk fibroin nanoparticle facilitates the lysosomal escape of nanoparticles, which avoids the degradation of nanoparticles in the lysosome. *Antheraea pernyi* silk fibroin nanoparticles also show multiple bio-responsive (pH, H_2_O_2_, and glutathione) release abilities. The hydrogen ion, H_2_O_2_, and glutathione reduce the β-sheet content in *Antheraea pernyi* silk fibroin nanoparticles, resulting in the unfolding of the protein structure, which promotes the release of resveratrol. Under the synergistic effect of many factors, resveratrol encapsulated in *Antheraea pernyi* silk fibroin nanoparticle was targeted to enterocytes repairing the epithelial barrier. The result shows that the ligand may exist in the natural material as a part, which can avoid the complex process of ligand grafting.

#### 4.1.4. Fucoidan-Based Targeting

Fucoidan, which is extracted from brown seaweed, contains repeating L-fucose in the main chain. There are some sulfate groups and minor monosaccharides in the side chain [56]. The fucoidan exhibits the capacity of enterocyte targeting due to the interaction between fucose and the fucose receptor. The soluble eggshell membrane protein has outstanding anti-oxidative and anti-inflammatory activities, but its application is limited due to the poor stability in the harsh gastrointestinal environment during oral administration. The chitosan/fucoidan nanoparticles were fabricated by ionic crosslinking and used to encapsulate the soluble eggshell membrane protein [35]. The release of the soluble eggshell membrane protein from nanoparticles exhibited pH dependence due to changes in the electrostatic interaction between chitosan and fucoidan, which resulted in the soluble eggshell membrane protein being stable in the stomach and released into the intestinal tract. The chitosan/fucoidan nanoparticles were effective in enterocyte-targeted delivery of soluble eggshell membrane protein, which was beneficial to the repair of epithelial defects. Edible materials may exhibit a dual capacity for nutraceutical loading and cell targeting, which is conductive to the accurate delivery of nutraceuticals and the simple development of carriers.

### 4.2. Macrophage Targeting

Macrophages, which are a kind of immune cell existing in different tissues, play an important role in maintaining health, such as clearing senescent or apoptotic cells and phagocytosing pathogens [57]. The macrophage can be polarized to the M1 or M2 macrophage at different stimuli. Interestingly, M1 and M2 macrophages play opposite roles, in which the M1 macrophage can trigger inflammation by secreting pro-inflammatory factors (IL-6, IL-12, and TNF-α) and the M2 macrophage can suppress inflammation by secreting anti-inflammatory factors (IL-10). Therefore, the inflammation can be mitigated by regulating the phenotype of the macrophage. In addition, a macrophage is closely related to tumor cells and can promote the movement and spread of tumor cells [58]. The delivery of nutraceuticals can be enhanced by improving the ability of macrophage targeting based on the ligand–receptor interaction. Ligands, which can be used for macrophage targeting, include HA [33], chondroitin sulfate (CS) [59], β-glucan [17], galactose [20], mannose [26], RGD [41], and methyl ester of phosphatidylglycerophosphate methyl ester (PGP-Me) [42].

#### 4.2.1. HA- or CS-Based Targeting

HA and CS belong to a kind of natural glycosaminoglycan. HA consists of alternating D-glucuronic acid and N-acetylglucosamine. CS consists of alternating D-glucuronic acid and N-acetylgalactosamine. They not only have functions of anti-inflammation and moisturization, but also can act as the ligand of a cluster of differentiation protein 44 (CD44) receptor expressed on macrophages, which can achieve the cell-targeted delivery of nutraceuticals [60]. In addition, the expression of CD44 on the macrophage in the inflammatory site is enhanced, which is beneficial to facilitating the macrophage targeting mediated by the ligand of HA or CS [61].

Magnolol is a kind of anti-inflammatory material extracted from *Magnolia officinalis*, but it has poor accumulation in an inflamed colon, limiting its therapeutic effects. Therefore, zein nanoparticles modified by CS were constructed through the antisolvent precipitation method and used for the targeted delivery of magnolol [22]. Notably, magnolol-loaded nanoparticles were encapsulated in sodium alginate/xanthan gum microparticles cross-linked by a calcium ion, which prolonged the colon retention time and led to the targeted release of nanoparticles in the colon. The magnolol encapsulated in the nanoparticles was transported to the macrophage, resulting in the down-regulation of pro-inflammatory cytokine levels, which showed an excellent therapeutic effect on ulcerative colitis. Curcumin, which is a natural diketone compound extracted from the rhizome of zingiberaceae, exhibits outstanding anti-inflammatory effects [62]. The curcumin-loaded silk fibroin nanoparticles functionalized by CS were used to relieve the symptoms of ulcerative colitis [38]. Furthermore, the curcumin-loaded silk fibroin nanoparticles were encapsulated in chitosan/alginate hydrogel to improve their gastrointestinal stability realizing the oral delivery. The results showed that the secretion of IL-10 was enhanced, alleviating the inflammation due to the fact that the curcumin was targeted to macrophages and released under the multi-bioresponsive conditions (pH, glutathione, and reactive oxygen species). CS can be used to construct cell-targeted delivery systems via covalent or non-covalent methods, which is beneficial to the delivery of nutraceuticals.

HA, as an excellent ligand, has also received extensive attention for cell-targeted delivery systems [63]. Rhein-loaded lactoferrin nanoparticles were modified by HA based on electrostatic interactions to improve the targeted ability of carriers and coated with calcium pectinate to enhance the gastrointestinal stability of carriers, which effectively alleviated ulcerative colitis by inhibiting the TLR4/MyD88/NF-κB pathway [33].

#### 4.2.2. β-Glucan-Based Targeting

β-glucan, which is composed of glucose linked by a β-glycosidic bond, can regulate the immunocompetence and the secretion of cytokines. For example, water-soluble yeast β-glucan could compete with lipopolysaccharide to bind with cell receptors, which inhibited mitogen-activated protein kinases and nuclear factor-kappa B signal pathways resulting in a reduction in pro-inflammatory factors (TNF-α, IL-6 and IL-1β) [64]. Furthermore, β-glucan can be used for ligand-specific binding with dectin-1 receptor expressed on macrophages to realize the targeted delivery of nutraceuticals [65]. Yeast cell microcapsules (YCMs) consist of β-glucan exhibiting the ability of macrophage targeting [66]. The cavity structure of YCMs is also conducive to the encapsulation of various nutraceuticals. In addition, YCMs can exist in macrophages for 7 days after endocytosis, which is beneficial to the long-term release of nutraceuticals [67]. Curcumin can be encapsulated in yeast glucan particles through solvent evaporation and ethanol is proved to be the most suitable solvent [40]. Furthermore, the encapsulation of curcumin has no influence on the ligand–receptor interaction and phagocytosis of macrophages, which indicates that the yeast glucan particles are outstanding cell-targeted delivery carriers. Emodin-loaded nanoparticles were encapsulated in the YCMs through electrostatic-force-driven auto-deposition [17]. YCMs could protect emodin-loaded nanoparticles from the attack of pepsin in the stomach. The cell experiment also showed that the uptake of macrophages to particles was enhanced due to the ligand–receptor interaction of β-glucan and dectin-1 [17]. In addition, the YCMs were used to encapsulate berberine and epigallocatechin gallate [39]. The nutraceuticals-loaded YCMs were transported to macrophages mediated by the β-glucan ligand, which transformed the M1 macrophage into an M2 macrophage showing excellent anti-inflammatory effects.

#### 4.2.3. Galactose-Based Targeting

Galactose is a monosaccharide consisting of six carbons and an aldehyde. It is the most rapidly absorbed monosaccharide in the intestinal tract. Moreover, galactose can be recognized by macrophage galactose-type lectins, which are overexpressed in the macrophages in the inflammatory site, due to the presence of a galactose recognition domain [3]. Natural exosome-like tea leaf nanoparticles, which were composed of protein, lipid, polysaccharide, polyphenol, and flavone, were successfully extracted from tea leaves by centrifugation [20]. Galactose was found in tea leaf nanoparticles, which meant that the nanoparticles might have the ability of macrophage targeting. The free galactose inhibited the cellular uptake of macrophages, which indicated that the tea leaf nanoparticles could be internalized by macrophages mediated by galactose. Furthermore, the results showed that the tea leaf nanoparticles could relieve the inflammation of the bowel due to the macrophage targeting of nanoparticles and the anti-inflammatory activity of epigallocatechin-3-gallate, epicatechin gallate, and quercetin-3-O-glucoside in nanoparticles. Natural cell-targeted particles isolated from foods are a promising therapeutic agent because of their outstanding biosafety and low cost. Much work is needed to find outstanding particles with cell-targeting ability and a treatment effect. The influence of the extraction method on the bioactive material of natural particles should be explored.

#### 4.2.4. Mannose-Based Targeting

Mannose as a ligand can be combined with the mannose receptor expressed on the macrophages to mediate the cell-targeted delivery of a carrier. The positively charged chitosan derivatives modified by mannose formed nanoparticles with negatively charged chitosan derivatives and bovine serum albumin (BSA) through electrostatic interaction [26]. The nanoparticles exhibited good stability in simulated gastric fluid and biosafety to cells, demonstrating their potential as an oral delivery system. Furthermore, the mucosal adhesion of chitosan derivative nanoparticles was enhanced due to the electrostatic interaction between positively charged nanoparticles and negatively charged mucin, which was beneficial to prolonging the residence time of nutraceuticals. Compared with nanoparticles without mannose, the nanoparticles decorated with mannose exhibited a higher macrophage uptake rate due to ligand–receptor interaction. The gastrointestinal stability, mucosal adhesion, and cell-targeting ability significantly improve the delivery ability of the carrier to nutraceuticals. In vitro digestion is not enough to simulate the complex process of in vivo digestion. Therefore, it is necessary to evaluate the digestion and absorption of delivery systems through in vivo models.

#### 4.2.5. PGP-Me-Based Targeting

PGP-Me is present in archaeolipids, which are extracted from archaebacteria *Halorubrum tebenquichense*. Moreover, PGP-Me is the ligand of scavenger receptor A1, which is expressed on macrophages [68]. Therefore, the nanoarchaeosomes fabricated by archaeolipids have the capacity of macrophage targeting. The nanoarchaeosomes are well resistant to enzyme attack due to the existence of PGP-Me, which can help the nanoarchaeosomes maintain stability until they reach the macrophages [69]. The superoxide dismutase (SOD), as a kind of protein, can be degraded by digestion enzymes, which limits the scavenging ability of reactive oxide species in the body. Nanoarchaeosomes, which exhibited the structure of nanovesicles, were fabricated by archaeolipids through the thin-film hydration and extrusion method to protect and deliver SOD [42]. Nanoarchaeosomes used as the carrier of SOD were targeted to macrophages mediated by PGP-Me, which avoided the degradation of SOD in the gastrointestinal tract. SOD-loaded nanoarchaeosomes completely inhibited the production of inflammatory cytokines (IL-6 and TNF-α) exhibiting outstanding anti-inflammatory activity. Furthermore, the SOD-loaded nanoarchaeosomes showed excellent storage stability for 5 months and could keep the enzyme activity during the storage, which was conducive to commercialization.

### 4.3. M Cell Targeting

Most M cells exist in the follicle-associated epithelium that overlays Peyer’s patches (PPs) and some exist in the side of villi [70]. M cells have a different structure to other cells. There is no mucus layer on M cells, which is conducive to the cell targeting of delivery systems. Furthermore, delivery systems can be transported into the lymphatic circulation through the M cells in PPs, which can avoid hepatic first-pass metabolism, resulting in an increase in nutraceutical bioavailability. M cells have a dome-like structure due to the invagination of basal plasma membranes, so the relevant immune cells, such as B lymphocytes, T lymphocytes, macrophages, and dendritic cells, can exist in the depression of M cells [71]. Although the structure of M cells is conducive to the uptake of targeted delivery systems, its number is few—less than 1% of the total epithelial cells. Therefore, the targeted delivery of M cells faces great challenges. The following describes oral CDSEMs for M cell targeting. The ligands, which are used for M cell targeting, include lectin [36] and β-glucan [37].

#### 4.3.1. Lectin-Based Targeting

Lectins first came to the public’s attention from raw kidney bean poisoning, which can cause abdominal pain and diarrhea due to the fact that they can be combined with the receptor of enterocytes and influence the function of enterocytes. However, lectin, as a kind of ligand, can endow delivery systems with M cell targeting based on the specific binding between the ligand and receptor [12,72]. *Ulex europaeus* agglutinin-1 (UEA-1) was conjugated to alginate through amide linkages mediated by 1-ethyl-3-[3 dimethylaminopropyl] carbodiimide [36]. The chitosan nanoparticles were fabricated by the crosslinking of tripolyphosphate followed by BSA absorption. The alginate-UEA-1 conjugate was used to coat the nanoparticles, which improved the stability of BSA in the gastric environment. Furthermore, the UEA-1 facilitated the M cell targeting of the delivery system mediated by the receptor of α-L-fucose. The delivery system induced systemic and mucosal immune responses. There are many kinds of lectins, such as tomato lectin, wheat germ agglutinin, and so on. They have great potential to build CDSEMs, which can promote the precise delivery of nutraceuticals to cells.

#### 4.3.2. β-Glucan-Based Targeting

Microparticles obtained from yeast are composed of β-glucan, which are generally recognized as safe. In addition, the hollow structure and the ligand characteristic of glucan particles are beneficial to the encapsulation and targeted delivery of nutraceuticals. Hollow glucan particles (2–4 μm) were extracted from baker’s yeast and used to encapsulate ovalbumin as a model antigen [37]. The results of transmission electron microscopy demonstrated that glucan particles were endocytosed by the transcellular pathway of M cells. The immune response was triggered after the particles were presented to T cells, resulting in a Th17-biased response. Therefore, the β-glucan is an effective ligand, which can be used to construct an M-cell-targeted delivery system to realize the precise release of nutraceuticals.

### 4.4. Dendritic Cell Targeting

The dendritic cell (DC), which is named for its dendritic or pseudopodia-like protrusions, is a kind of antigen-presenting cell with the capacities of absorbing, processing, and presenting antigens. DCs can differentiate into an activated phenotype or tolerogenic phenotype under different stimuli, which can cause inflammatory or anti-inflammatory adaptive immunity [73]. DC targeting has become an effective strategy to solve health problems, which can be used for the treatment of malignant tumors [74]. Exosome-like nanoparticles extracted from edible plants exhibit outstanding biosafety and have good therapeutic effects on different diseases [75]. Broccoli-derived nanoparticles (BDNs) were isolated from edible plants and are rich in sulforaphane [43]. The BDNs were uptaken by DC in mesenteric lymph nodes and the colon, but the ligand and receptor were not elucidated. BDNs had an excellent preventive effect on colitis, which was due to the fact that they induced the formation of tolerogenic DC by the activation of adenosine-monophosphate-activated protein kinase. The natural exosomes are composed of many components, some of which may have the function of a ligand making them have the potential of cell-targeted delivery.

### 4.5. Tumour Cell Targeting

The regulation of tumor cell proliferation and differentiation is abnormal, which generates a great threat to health and life. Many receptors are expressed on the surface of tumor cells to absorb nutrients to maintain cell proliferation, which provides the conditions for the construction of cell-targeted delivery systems. The following describes oral CDSEMs for tumor cell targeting. The ligands, which are used to modify the delivery systems, include folic acid [46] and fucoidan [44].

#### 4.5.1. Folic Acid-Based Targeting

Folic acid is a kind of water-soluble vitamin which can bind with the folic acid receptor expressed on cells to achieve a cell-targeting effect [76,77]. Furthermore, the results of studies showed that the folic acid could reduce the activity of α-amylase, pepsin, and trypsin by forming complexes with enzymes, which could avoid the degradation of CDSEMs before reaching specific cells [78,79]. Folic acid–amino chitosan nanoparticles were fabricated through the ionic crosslinking of tripolyphosphate and used to encapsulate curcumin [45]. In vitro cell experiments showed that the uptake of nanoparticles by tumor cells was enhanced due to the presence of folic acid ligand. The amylopectin–albumin nanogels modified by folic acid were successfully constructed to transport curcumin [47]. The nanogels not only could resist the enzymatic hydrolysis in the gastrointestinal tract due to the starch shell formed by regeneration, but also promoted the internalization of cancer cells based on the ligand–receptor interaction. In addition, the curcumin, as a medium, was used to connect *Bacillus coagulans* spores and folic acid to construct cell-targeted delivery systems [46]. Less curcumin was released in the stomach because the spores were dormant, which increased the stability of curcumin in the harsh environment of the stomach. During intestinal digestion, the outer coat protein of the *Bacillus coagulans* spores was detached following the germination of the spores. The curcumin–folic acid complex linked with the outer coat protein self-assembled into nanomicelles, which were targeted to colon cancer cells mediated by folic acid. The spore-based delivery system significantly improved the oral bioavailability of curcumin and effectively decreased the tumor size depending on cell targeting mediated by folic acid.

#### 4.5.2. Fucoidan-Based Targeting

Fucoidan, as a natural polysaccharide, can combine with the P-selectin receptor, which is overexpressed in tumor cells [80]. In addition, fucoidan exhibits anti-cancer activity. Therefore, fucoidan-based delivery systems are fabricated to treat pancreatic cancer. The dual-targeted nanoparticles were fabricated by fucoidan and lactoferrin based on electrostatic interaction, in which the fucoidan and lactoferrin were used to combine with the P-selectin receptor and lactoferrin receptor overexpressed in cancer cells, respectively [44]. The targeting properties and anti-cancer activity of fucoidan effectively inhibited the migration of cancer cells. The results show that the edible material can play a dual function of targeting and therapy to maintain health.

## 5. Future Perspectives

**Figure 4:**
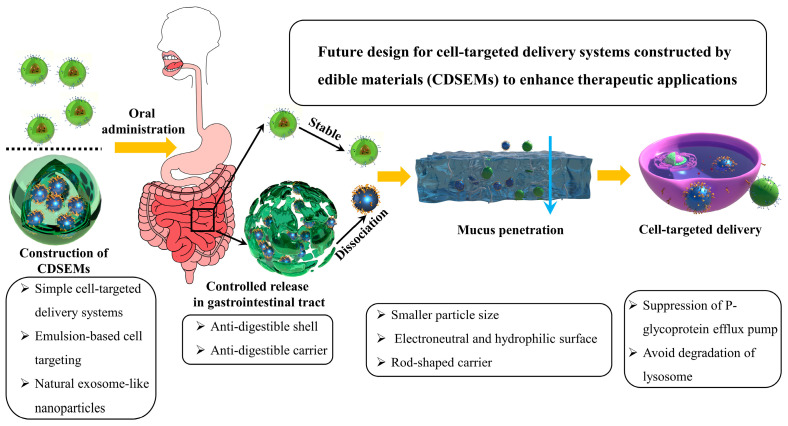
Multi-angle approaches for the development of cell-targeted delivery systems constructed of edible materials (CDSEMs) for advanced delivery.

In the past few decades, oral cell-targeted delivery, as an effective method, has significantly enhanced the precise delivery ability of nutraceuticals in the body and exhibits great potential in the field of health. Many edible materials are used to construct cell-targeted delivery systems, which show outstanding biosafety. Compared with delivery systems fabricated using synthetic materials, the performance of CDSEMs should be further improved in the following aspects: enhancing gastrointestinal stability, overcoming the mucosal barrier, and improving bioavailability. Future perspectives are given below to enhance cell-targeted delivery for CDSEMs (Figure 4).

### 5.1. Simple Cell-Targeted Delivery Systems

Delivery systems mainly rely on ligand–receptor interaction to achieve the goal of cell targeting. The ligands are generally vitamins, proteins, and polysaccharides, which are generally recognized as safe materials. In general, the ligand should be grafted on the delivery carrier, but the process of grafting might use crosslinking agents, such as EDC. The use of a chemical crosslinking agent not only increases the production cost but also may raise concerns about safety. The method of the Maillard reaction or enzymatic crosslinking can be considered to graft the ligand onto the delivery carriers, which can avoid the introduction of other chemical reagents. In addition, studies have shown that natural substances contain fragments with a targeting function, such as RGD of *Antheraea pernyi* silk fibroin, β-glucan of yeast microcapsules, PGP-Me of archaeolipids, and fucose of fucoidan, which can be designed as cell-targeted delivery systems avoiding the grafting step of ligands [35,39,41,42]. Therefore, it requires a great amount of work to analyze the fine structure of edible materials to provide the basis for the development of natural carriers with targeting ability.

### 5.2. Emulsion-Based Cell Targeting

An emulsion consists of two incompatible phases and emulsifiers, which are adsorbed on the interface of the two phases to keep the stability of the emulsion [81]. Emulsions have been widely used as delivery carriers of nutraceuticals and have significantly improved their stability and bioaccessibility [82]. For instance, the Pickering emulsion stabilized by ovalbumin fibril improved the astaxanthin bioaccessibility due to high specific surface area [83]. Furthermore, peptides-loaded nanoemulsions have been ingested by intestinal cells and transported into the pancreas through intestinal lymphatic transport, indicating that emulsions could be used as a carrier of cell-targeted delivery [84]. There are few reports on the cell targeting of emulsions. Emulsifiers located at the interface can be the grafting sites of ligands to endow an emulsion with the ability of cell targeting. However, the emulsifier will rearrange at the oil–water interface after it is absorbed at the interface [85]. In the process of emulsifier rearrangement, the change in the ligand has not been expounded. It remains to be investigated whether ligands with different structures will lose their ability to bind with the receptor after the rearrangement of emulsifiers. In addition, the ligand located at the oil–water interface means that it is firstly attacked by the digestive enzymes, which also leads to the loss of targeting ability. Therefore, protection for the emulsion might be necessary before the carrier is delivered to the targeted cell. Calcium-pectinate-based emulsion gel can be constructed, which leads to the release of the emulsion in the colon. Furthermore, the ligand located at the oil–water interface can promote cell targeting.

### 5.3. Digestive Stability of the Delivery Systems

Delivery systems constructed of edible materials have excellent biocompatibility and biodegradability, which means that they can be hydrolyzed by various enzymes in the gastrointestinal tract. This is one of the challenges faced by oral CDSEMs. It is very important for the delivery system to maintain its integrity before reaching specific cells, which can avoid the premature release of cargo. First of all, it is necessary to clarify the location of the targeted cells, such as in the small intestine or the large intestine. After that, anti-digestible materials, such as resistant starch, pectin, alginate, inulin, yeast cell microcapsules, and so on, are selected to protect the targeted delivery systems from disassembly in the harsh gastrointestinal environment [63,86,87,88]. Furthermore, the structural and functional properties of various ligands should be fully understood. For example, folic acid as a ligand can inhibit the activity of α-amylase, pepsin, and trypsin, which not only endows delivery systems with the capacity of cell targeting but also prevents the degradation of delivery systems before reaching the cells [78,89]. Further analysis of the protective effect of folic acid grafted on the delivery system during oral administration is helpful to simplify the design of oral CDSEMs, which can avoid the use of additional protective layers. Surface layer protein B from *Levilactobacillus brevis* also has the function of targeting and improving the gastrointestinal stability of delivery systems [90]. Therefore, it is necessary to further develop and design anti-digestible carriers to keep the integrity of delivery systems before reaching the targeted cells. Furthermore, some ligands must be processed in the gastrointestinal tract to play a targeted role, which means that the protective layer is not applicable [31]. For example, VB_12_ must bind to IF in the small intestine to form a complex, which can be combined with the IF receptor of cells and mediates endocytosis [91].

### 5.4. Mucus Penetration Ability

The mucus layer, which is located at the apical surface of cells, is the last barrier for targeted delivery systems arriving at the cells mediated by the ligand. The mucus layer consists of mucins, lipids, and even food residues. The negatively charged mucins inhibit the penetration of the delivery systems through electrostatic interactions. The mucins and lipids can also hinder mucus penetration through hydrophobic interactions [25]. The muco-inert nanoparticles with an electroneutral and hydrophilic surface have an enhanced ability of mucus penetration. Although polyethylene glycol can be an excellent coating to enhance the penetration of nanocarriers, it causes the oxidation of nutraceuticals and an allergic reaction of the body [14,15]. Therefore, it is necessary to develop oral CDSEMs with outstanding mucus penetration. Some viruses exhibit outstanding penetration capacity to mucus due to the densely charged surface, which can be used to design a biomimetic structure through edible materials with opposite charge to enhance the mucus penetration of the carriers [92]. The outer material should be hydrophilic to prevent the hydrophobic interaction between the carrier and the mucus layer.

The size and morphology of cell-targeted delivery systems also affect the ability of mucus penetration. The aperture of the three-dimensional net structure of the mucus layer is about 10–200 nm, so smaller nanocarriers can penetrate the mucus layer more easily. Additionally, most of the existing nanocarriers are spherical, but research has shown that the rod-shaped carriers have superior mucus penetration ability, which is due to the improvement in rotational dynamics of the nanocarriers [93]. The rod-shaped carriers are beneficial to the uptake of intestinal cells [94]. However, there are few studies on rod-shaped carriers prepared from edible materials. Therefore, the mucosal penetration ability of CDSEMs should be enhanced through the design of the carrier in size, morphology, surface charge, and hydrophilicity/hydrophobicity.

### 5.5. Dual-Ligand Modification

The modification of the ligand in the delivery system significantly enhances the delivery of nutraceuticals. However, the modification of single ligand might be not enough to overcome the complex physiological barriers [95]. For example, the carrier cannot be combined with a macrophage before it passes through the epithelial cells. In addition, time is needed for the recycling and synthesis of the receptor, which might limit the ligand–receptor interaction. A variety of receptors are expressed on the cell membrane. Therefore, dual-ligand-modified delivery systems have attracted the attention of researchers [96,97]. The two ligands may mutually interfere through electrostatic interaction, which has a negative influence on cellular uptake [98]. In general, the density, ratio, and length of two ligands are regulated to improve the cell-targeting ability of a carrier [99]. In the latest research, ligand-switchable nanoparticles were fabricated to overcome the mutual interference between two ligands enhancing the delivery ability of the carrier [100]. Cell-penetrating peptide and galactose were simultaneously used to modify the delivery system, in which the cell-penetrating peptide showed pH-responsive stretchability. The cell-penetrating peptide can extend at pH 6.8, which makes nanoparticles traverse intestinal barriers. The cell-penetrating peptide can fold at pH 7.4, leading to the exposure of galactose at the surface of nanoparticles, which makes nanoparticles reach the liver through specific targeting. In CDSEMs, dual-ligand modification might be an excellent method to enhance the cell-targeting delivery of nutraceuticals. However, the type, ratio, and length of the ligand should be carefully considered to avoid the mutual interference between dual ligands.

### 5.6. Hepatic First-Pass Metabolism

The CDSEMs are transported to specific cells mediated by ligands, which does not mean that they can play a role. If the nutraceuticals are expected to be transported into the systemic circulation through oral administration, it is necessary to consider the effect of hepatic first-pass metabolism [101]. Hepatic first-pass metabolism refers to the metabolism of nutraceuticals in the liver before they enter the systemic circulation through oral administration, which decreases the bioavailability of nutraceuticals. The results of research showed that water-soluble substances were transported into portal circulation. The fat-soluble substances were transported into the intestinal lymphatic system, which could bypass hepatic first-pass metabolism [70]. There are many nutraceuticals belonging to fat-soluble substances, such as astaxanthin, fucoxanthin, α-tocopherol, quercetin, and so on, having the potential to be transported into the intestinal lymphatic system. Fat-soluble nutraceuticals can be encapsulated in lipid carriers, which are processed into chylomicrons and transported into the intestinal lymphatic circulation as they pass through intestinal epithelial cells. The 5-demethylnobiletin encapsulated in canola oil emulsion was processed to chylomicrons in a Caco-2 cell, which promoted the lymphatic transport of 5-demethylnobiletin [102].

### 5.7. Oral Effectiveness

The cell-targeted delivery of nutraceuticals by oral administration is a desirable approach, but oral effectiveness should be further considered. Oral administration may need to be compared with injection to ensure oral effectiveness. The targeted delivery capacity of a biotin-modified nanomicelle was evaluated by intravenous administration and oral administration [32]. The nanomicelle could enter the systemic circulation and exist for 48 h through intravenous administration, but it could not enter the systemic circulation and was eliminated through oral administration. The nanomicelle was excreted and eliminated with the intestinal content, which was due to the activation of the P-glycoprotein efflux pump during oral administration [32]. Therefore, the role of P-glycoprotein should be considered when the oral bioavailability of cell-targeted delivery systems is poor. Furthermore, the therapeutic effects of curcumin-loaded nanoparticles modified by CS on ulcerative colitis were compared by oral administration or intravenous injection [38]. The results showed that intravenous injection was better than oral administration, which was due to the fact that the curcumin-loaded nanoparticles were eliminated with feces during oral administration [38]. Therefore, the therapeutic effects of oral and injection administrations should be compared, so as to give full play to oral advantages and ensure oral therapeutic effects.

## 6. Conclusions

In conclusion, cell-targeted delivery systems can be entirely constructed of edible materials, which exhibit excellent biosafety. Some edible materials, such as resistant starch, calcium pectinate, alginate, and yeast cell microcapsules, can be used as the protective shell to resist the harsh gastrointestinal environment, avoiding the premature degradation of cell-targeted carriers. The mucosal barrier can be overcome by regulating the hydrophilicity/hydrophobicity, surface charge, and morphology of the carrier. However, from the perspective of the selection of material, the targeting of the cell, and the release of nutraceuticals, there is still room for development and improvement in oral CDSEMs to effectively solve health problems. The carrier structure should be further designed to avoid mucosal clearance, lysosomal degradation, and hepatic first-pass metabolism, increasing the efficacy of nutraceuticals in the targeted site. In addition, cell-targeted delivery systems with a simple structure should be developed, which can reduce the use of chemical reagents and the cost of production. In-depth analysis of the composition and structure of edible materials is also conducive to the development of cell-targeted delivery systems. Sustaining effort and improvement in the fields of food and biology are expected to bring oral CDSEMs to market.

## Figures and Tables

**Table 1:** The structure and function of cell-targeted delivery systems constructed of edible materials.

Cell	Ligand	Receptor	Cargo	Delivery System	Cell/ Animal Model	Result	Ref.
Enterocyte	VB_12_	Intrinsic factor receptor	Scutellarin	VB_12_-amphiphilic chitosan nanoparticles	Caco-2/Rat	The transport volume of scutellarin increased 4.39 times.	[31]
	Biotin	SMVT	–	Nanomicelle assembled from inulin–vitamin E conjugate modified by biotin	Caco-2/Mice	The activation of the efflux pump decreased the oral bioavailability.	[32]
	Lactoferrin	Lactoferrin receptor	Rhein	Lactoferrin nanoparticles modified by calcium pectinate and HA	RAW 264.7 and Caco-2 /Mice	The nanoparticles were targeted to enterocytes and up-regulated the expression of ZO-1 and Claudin-1.	[33]
	Lactoferrin	Lactoferrin receptor	–	Zein/lactoferrin nanoparticles	Caco-2/Rat	Cell uptake was enhanced through the recognition of lactoferrin receptors and electrostatic interaction of nanoparticles with negatively charged glycocalyx.	[34]
	Fucoidan	Fucose receptor	Soluble eggshell membrane protein	Chitosan/fucoidan nanoparticles	Caco-2 and RAW 264.7	Soluble eggshell membrane protein and nanoparticles synergistically increased the anti-oxidant activity.	[35]
M cell	UEA-1	α-L-fucose	Bovine serum albumin	Chitosan nanoparticles coated in alginate-UEA-1 conjugate	-/Mice	The particles induced systemic and mucosal immune responses.	[36]
	β-glucan	–	Ovalbumin	β-glucan microparticles	Caco-2 and HT-29/Mice	The microparticles could be transported by an M cell by the transcellular pathway and triggered an immune response.	[37]
Macrophage	CS	CD44 receptor	Curcumin	CS-decorated silk fibroin nanoparticles encapsulated in chitosan/alginate hydrogel	RAW 264.7/Mice	Macrophage targeted delivery and multiple bio-responsive releases of curcumin enhanced its therapeutic effect.	[38]
	CS	CD44 receptor	Magnolol	CS-decorated zein nanoparticles encapsulated in sodium alginate/ xanthan gum microspheres	RAW 264.7 and NCM 460 /Mice	Alginate/xanthan gum microspheres prolonged the colon retention of nanoparticles and the targeted release of magnolol decreased the levels of pro-inflammatory cytokines.	[22]
	β-glucan	Dectin-1 receptor	Berberine and EGCG	Yeast microcapsule	RAW 264.7 and L929 /Mice	Berberine and EGCG encapsulated in yeast microcapsules were targeted to the macrophage and showed synergistic anti-inflammatory effects.	[39]
	β-glucan	Dectin-1 receptor	Emodin	Yeast cell wall microparticles	RAW 264.7 and Caco-2/Mice	Gastrointestinal tract stability and the targeted ability of nanoparticles were enhanced by yeast cell wall microparticles resulting in the enhancement of the anti-inflammatory effects of emodin.	[17]
	β-glucan	–	Curcumin	Yeast glucan particles	J774A.1/-	The encapsulation of curcumin had no influence on the phagocytosis of macrophages.	[40]
	Galactose	Galactose receptor	Polyphenols and flavones	Tea-leaf-derived nanoparticles	RAW 264.7/Mice	Nanoparticles derived from tea leaf showed targeted ability and inhibited intestinal inflammation.	[20]
	Mannose	Mannose receptor	Bovine serum albumin	Chitosan derivative nanoparticles	RAW 264.7/-	The nanoparticles showed good mucosal adhesion and were uptaken by macrophages.	[26]
	RGD	Integrin receptor	Resveratrol	*Antheraea pernyi* silk fibroin nanoparticles	RAW 264.7 and CT-26/Mice	The nanoparticles polarized macrophages to type M2 and relieved symptoms of ulcerative colitis.	[41]
	PGP-Me	Scavenger receptor A1	Superoxide dismutase	Nanoarchaeosomes (nanovesicles)	J774A.1 and Caco-2/-	The cellular uptake capacity for nanoparticles increased by 6.4 times.	[42]
Dendritic Cell	–	–	Sulforaphane	Broccoli-derived nanoparticles	-/Mice	Broccoli-derived nanoparticles containing sulforaphane activated AMPK to maintain intestinal immune homeostasis by dendritic cell targeting.	[43]
Tumor cell	Fucoidan and lactoferrin	P-selectin receptor/lactoferrin receptor	Fucoidan	Fucoidan/lactoferrin nanoparticles	PANC-1/-	IC_50_ value decreased by 2.3 folds.	[44]
	FA	–	Curcumin	FA-amino chitosan nanoparticles	LS174T/-	The cellular viability rate was less than 40% when the curcumin concentration was 25.0 μg/mL.	[45]
	FA	FA receptor	Curcumin	FA-*Bacillus coagulans* spore complex	HT-29/Rat	The assembly of folic-acid-grafted nanomicelles was promoted by the germination of spores realizing the cell-targeted delivery in the colon.	[46]
	FA	FA receptor	Curcumin	FA-amylopectin– albumin core–shell nanogels	HT-29 and A549/-	The nanogels were resistant to digestion and enhanced the apoptosis rate of cancer cells as a carrier of curcumin.	[47]

–, not applicable; FA, folic acid; VB_12_, vitamin B_12_; HA, hyaluronic acid; CS, chondroitin sulfate; RGD, arginine–glycine–aspartate; PGP-Me, phosphatidylglycerophosphate methyl ester; EGCG, epigallocatechin gallate; SMVT: sodium-dependent multivitamin transporter; UEA-1, *Ulex europaeus* agglutinin-1; CD44, cluster of differentiation protein 44; AMPK, adenosine-monophosphate-activated protein kinase.

## Data Availability

Not applicable.
